# Stool energy density is positively correlated to intestinal transit time and related to microbial enterotypes

**DOI:** 10.1186/s40168-022-01418-5

**Published:** 2022-12-12

**Authors:** Jos Boekhorst, Naomi Venlet, Nicola Procházková, Mathias L. Hansen, Christian B. Lieberoth, Martin I. Bahl, Lotte Lauritzen, Oluf Pedersen, Tine Rask Licht, Michiel Kleerebezem, Henrik M. Roager

**Affiliations:** 1 Host-Microbe Interactomics, Wageningen University and Research, Wageningen, The Netherlands; 2 Department of Nutrition, Exercise and Sports, University of Copenhagen, Frederiksberg, Denmark; 3 National Food Institute, Technical University of Denmark, Kgs. Lyngby, Denmark; 4 The Novo Nordisk Foundation Center for Basic Metabolic Research, University of Copenhagen, Copenhagen, Denmark

**Keywords:** Microbial ecology, Intestinal transit time, Energy harvest, Personalised nutrition

## Abstract

**Background:**

It has been hypothesised that the gut microbiota causally affects obesity via its capacity to extract energy from the diet. Yet, evidence elucidating the role of particular human microbial community structures and determinants of microbiota-dependent energy harvest is lacking.

**Results:**

Here, we investigated whether energy extraction from the diet in 85 overweight adults, estimated by dry stool energy density, was associated with intestinal transit time and variations in microbial community diversity and overall structure stratified as enterotypes. We hypothesised that a slower intestinal transit would allow for more energy extraction. However, opposite of what we expected, the stool energy density was positively associated with intestinal transit time. Stratifications into enterotypes showed that individuals with a *Bacteroides* enterotype (B-type) had significantly lower stool energy density, shorter intestinal transit times, and lower alpha-diversity compared to individuals with a *Ruminococcaceae* enterotype (R-type). The *Prevotella* (P-type) individuals appeared in between the B- and R-type. The differences in stool energy density between enterotypes were not explained by differences in habitual diet, intake of dietary fibre or faecal bacterial cell counts. However, the R-type individuals showed higher urinary and faecal levels of microbial-derived proteolytic metabolites compared to the B-type, suggesting increased colonic proteolysis in the R-type individuals. This could imply a less effective colonic energy extraction in the R-type individuals compared to the B-type individuals. Notably, the R-type had significantly lower body weight compared to the B-type.

**Conclusions:**

Our findings suggest that gut microbial energy harvest is diversified among individuals by intestinal transit time and associated gut microbiome ecosystem variations. A better understanding of these associations could support the development of personalised nutrition and improved weight-loss strategies.

Video Abstract

**Supplementary Information:**

The online version contains supplementary material available at 10.1186/s40168-022-01418-5.

## Background

More than a decade ago studies on rodents indicated that the gut microbiota may influence host energy balance [1, 2], and it was proposed that an obese-associated gut microbiota influence host physiology through increased capacity for harvesting energy from the diet [3]. Following these remarkable observations, several studies confirmed that when transplanting an obese-associated gut microbiota into gnotobiotic mice, the mice indeed gained more body weight or fat mass compared to mice transplanted with a lean-associated gut microbiome [4–6]. This notion was corroborated by the finding that the weight gain of human-microbiota transplanted gnotobiotic mice was negatively correlated with faecal gross energy [3, 5], implying that differences in the gut microbiome’s capacity to extract energy from the diet could be relevant for weight management. Originally, the ratio between the two dominant phyla of the gut microbiota, the *Firmicutes* and *Bacteroidetes*, was suggested to be a marker of obesity [7]. Yet, the proportion of the *Firmicutes*/*Bacteroidetes* ratio did not correlate with energy harvest markers [8] and is today no longer considered a relevant marker of obesity [9]. Instead, stratification into microbial enterotypes according to microbial community structures with marked abundance of either *Prevotella* (P-type), *Bacteroides* (B-type) or *Ruminococcaceae* (R-type), respectively [10, 11], could be of relevance since these appear to be robust across the world [12] and have been reported to be overall stable in individuals over time [13]. Furthermore, these enterotypes have been suggested to differ in metabolic capacity for degradation of carbohydrates, proteins, and lipids [14]. However, it remains unknown whether enterotypes differ in their capacity to harvest energy from the diet. Moreover, it is not known whether intestinal transit time, which is linked to gut microbial composition, diversity, and metabolism [15–19], is a determinant of microbiota-dependent energy harvest in the gut. Therefore, we investigated whether gut microbial energy extraction, as reflected by dry stool energy density, was associated with intestinal transit time and microbiome community structures in 85 overweight adults.

## Methods

### Study and subjects

**Fig. 1:**
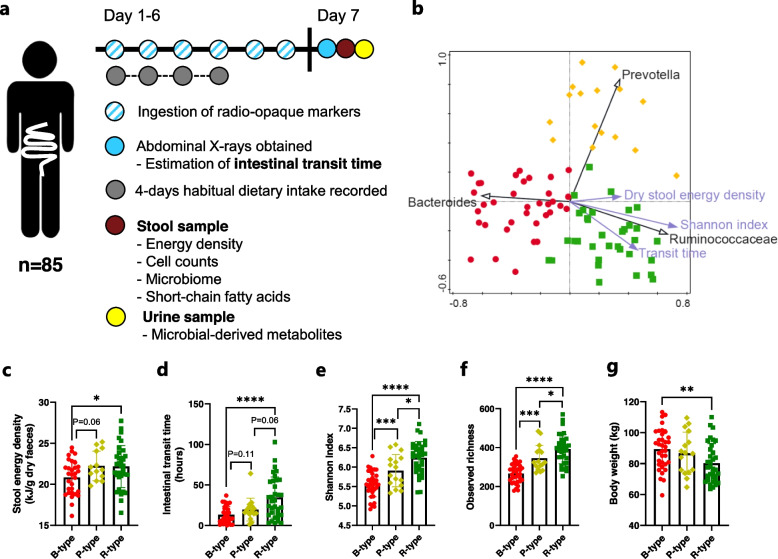
Enterotypes differ in stool energy density, intestinal transit time, microbial alpha-diversity, and body weight. **a** The study included baseline measurements of 85 overweight subjects. Prior to collection of the stool and urine samples used in the study, habitual dietary intake was estimated based on 4-day dietary registrations and intestinal transit time was estimated using radio-opaque markers from day 1 to 6 where participants maintained their habitual diet and lifestyle. The collected stool sample was used to estimate dry stool energy density as a measure of gut microbial energy extraction, bacterial cell counts, the gut microbiome community structure, and short-chain fatty acids. Microbial-derived metabolites were measured in the urine samples. **b** Principal coordinate analysis plot using Bray-Curtis distance of bacterial relative abundance on the genus level as distance metric. Symbols are samples, with shape/colour indicating assigned enterotype (red circles: *Bacteroides* (B-type), *n* = 35; yellow diamonds: *Prevotella* (P-type), *n* = 16; green squares: *Ruminococcaceae* (R-type), *n* = 34). Relative abundance of the taxa used for enterotype assignment (black arrows) and values for dry energy, Shannon index and transit time (purple arrows) were plotted supplementary (i.e. projected after ordination). Horizontal and vertical axis explain 20% and 12% of variation, respectively. Subjects stratified into three enterotypes differed in **c** stool energy density (*n* = 77), **d** intestinal transit time (*n* = 85), microbiome alpha-diversity as reflected by **e** Shannon Index and **f** observed richness (*n* = 85), as well (**g**) body weight (*n* = 85). Differences between enterotypes were detected using the Mann-Whitney *U* test. *p < 0.05, **p < 0.01, ***p < 0.001, ****p < 0.0001

In total, baseline data and samples collected from 85 subjects (53 female and 32 male, aged 22–66 years, median of 52) (Fig. 1a), exhibiting an increased metabolic risk, who participated in two human intervention studies within the Gut, Grain and Greens (3G) Center [20], were included in the present study (Table 1). The participants were weight stable with a body mass index of 25–35 kg/m^2^ and/or increased waist circumference (≥ 94 cm for men and ≥ 80 cm for women). Additionally, they fulfilled at least one of the following criteria: non-diabetic dysglycaemia (fasting plasma glucose 6.1–6.9 mmol/L), dyslipidaemia (fasting serum high-density lipoprotein (HDL) cholesterol ≤ 1.03 mmol/L for men and ≤ 1.29 mmol/L for women) or hypertension (systolic blood pressure (BP) > 130 mm Hg or medical treatment of hypertension). Exclusion and inclusion criteria have previously been described in detail [21, 22]. Participants did not take food supplements or drugs affecting intestinal transit time, and physical activity was not monitored. See flow chart of participants and data in Supplementary Figure S1.

**Table 1:** Characteristics of participants (values are presented as mean ± SD, *n* = 85)

Characteristics	*All*	*B-type*	*P-type*	*R-type*
**Body composition**				
Sex (F/M)	53/32	21/14	8/8	24/10
Age (years)	49.8 ± 11.2	50.1 ± 10.4	48.3 ± 12.6	50.3 ± 11.1
Body weight (kg)	85.2 ± 13.3	89.3 ± 12.5	86.6 ± 13.2	80.2 ± 12.4
**Glucose metabolism**				
Fasting plasma glucose (mmol/L)	5.7 ± 0.6	5.8 ± 0.5	5.5 ± 0.7	5.7 ± 0.7
Fasting serum C-peptide (pmol/L)	792 ± 237	834 ± 229	698 ± 201	793 ± 247
Fasting serum insulin (pmol/L)	63.2 ± 29.9	72.3 ± 33.4	48.1 ± 13.4	60.9 ± 28.4
**Lipids**				
Fasting serum total cholesterol (mmol/L)	5.3 ± 0.9	5.4 ± 0.8	5.3 ± 1.0	5.1 ± 0.9
Fasting serum LDL cholesterol (mmol/L)	3.1 ± 0.7	3.2 ± 0.7	3.2 ± 0.9	3.0 ± 0.7
Fasting serum HDL cholesterol (mmol/L)	1.3 ± 0.3	1.3 ± 0.3	1.4 ± 0.3	1.3 ± 0.3
**Blood pressure**				
Systolic BP (mmHg)	85 ± 43	92 ± 42	93 ± 42	75 ± 43
Diastolic BP (mmHg)	64 ± 20	68 ± 18	69 ± 17	59 ± 22

*LDL* low-density lipoprotein, *HDL* high-density lipoprotein, *BP* blood pressure

### Sample collection and measurements of habitual diet and intestinal transit time

For detailed experimental procedures and data analyses, we refer to our previously published paper [16]. In brief, each participant recorded their habitual dietary intake by a 4-day precoded dietary registration prior to the examination day on two weekdays and two weekend days [20]. The questionnaire was organised according to the typical Danish meal pattern (breakfast, lunch, dinner and snacks). Daily intake of total energy, macronutrients and certain food components and food groups were calculated using the Danish Food Composition Databank [23]. Furthermore, food intake was adjusted by total energy intake for each participant (g/day/kJ). Intestinal transit time was estimated using radio-opaque markers, which was ingested for six consecutive days (day 1–6) before the examination day (day 7), as published before [16]. During these six days, the participants maintained their habitual diet and lifestyle. On the examination day (day 7), each participant delivered a stool sample in a plastic bag, which was stored at 5 °C for maximally 24 h, then homogenised in sterile water 1:1, aliquoted and stored at – 80 °C until further processing. Furthermore, the participants arrived in morning and were weighted, had their blood pressure measured, and a fasting blood sample was drawn. The blood sample was analysed for concentrations of glucose, insulin, C peptide, total cholesterol, LDL cholesterol, and HDL cholesterol as previously published [21, 22]. In addition, a urine sample was collected for 4 h in a standardised way following a standardised drink and breakfast on the examination day as previously published [21, 22].

### Stool energy density, cell counts, and microbiome characterisation

An aliquot of the homogenised stool sample (1 mL) was dried at 50 °C in an oven for 72 h or until dry. Subsequently, the dry stool material was weighed using a four-decimal scale (AG204 Delta Range). Gross energy density of stool samples (*n* = 80) was determined by combusting ∼ 100 mg of dry stool material in a bomb calorimeter C6000 (IKA, Staufen, Germany) using benzoic acid as a calibrator (C723 IKA). Stool bacterial cell counts and the gut microbiome compositions have previously been published [16, 24]. In brief, an aliquot of stool sample (*n* = 83) was used to estimate bacterial cell counts by flow cytometry analysis [24], and an aliquot of stool (*n* = 85) was used to extract microbial DNA, amplify the V3–V4 region of the 16S rRNA gene, and sequence the gut microbiota on the Illumina MiSeq platform as previously published [16]. The microbiome sequencing data were rarefied to 21,000 reads per sample and the microbiome composition at genus level and alpha diversity (Shannon index and observed richness) were calculated using QIIME as previously published [16]. Enterotypes were assigned based on sample position in the principal coordinate analysis (PCoA) plot with the taxa characteristic for the three enterotypes plotted as supplementary variables. Sequence analysis data were deposited in the NCBI SRA database under accession no. SRP070699

### Urine metabolomics and faecal short-chain fatty acids

The urine metabolome data have previously been published [16]. In brief, all collected urine samples were profiled by liquid chromatography mass spectrometry and microbiota-derived protein degradation products (p-cresol sulfate, p-cresol glucuronide, and phenylacetylglutamine) were identified. Faecal short-chain fatty acids (SCFAs) were quantified by targeted liquid chromatography mass spectrometry as previously described [25]. In brief, SCFAs were quantified in faecal samples in two different dilutions (1:240 and 1:2400 dilution, respectively). Homogenised faecal samples were derivatised using 3-nitrophenylhydrazine (3NPH). Derivatised ^13^C_6_-SCFA-analogues (acetic acid, propionic acid, butyric acid, isobutyric acid, 2-methylbutyric acid, isovaleric acid, valeric acid, and caproic acid, respectively) were produced and used as isotope-labelled internal standards. The samples were randomised and analysed by a UPLC-QTOF-MS (Waters) in negative ionisation mode as previously reported [25]. The raw UPLC-MS data were analysed using QuanLynx (Waters Corporation). The calibration curves were established by plotting the peak area ratios between the individual SCFA analytes and labelled internal SCFA standards against the concentrations of the calibration standards. The calibration curves were fitted to a linear regression. The average R2 of all external standard calibration curves was 0.98.

### Statistical analyses

Statistical analyses were conducted in GraphPad Prism (version 9.1.0). Stool energy density outliers (*n* = 3) were identified using the ROUT method with a maximum desired false discovery rate set to 1% [26]. Correlations were calculated using Spearman’s rank correlation. Differences between enterotypes were detected using the Mann-Whitney *U* test. A *p* value below 0.05 was considered statistically significant. PCoA was done in Canoco version 5.12 [27] with default settings, with missing data for supplementary variables replaced with the corresponding median value.

## Results

**Fig. 2:**
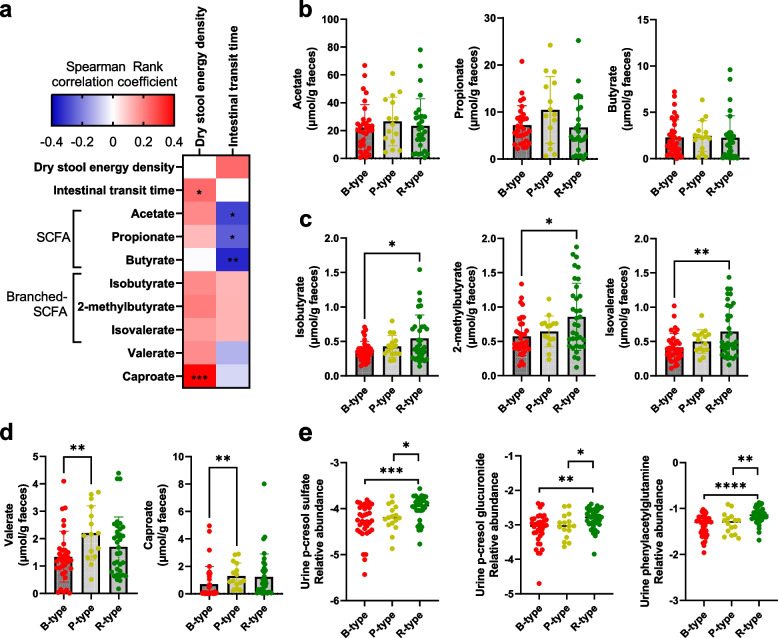
Enterotypes differ in proteolytic metabolites in faeces and urine. **a** Heatmap showing Spearman correlation coefficients of the associations between dry stool energy density, intestinal transit time and faecal short-chain fatty acids (SCFAs). Faecal concentration of SCFAs according to the three enterotypes with respect to **b** the SCFAs acetate, propionate and butyrate, **c** the branched SCFAs isobutyrate, 2-methylbutyrate, and isovalerate, as well as **d** valerate and caproate. **e** Log-transformed urinary relative levels of the microbial-derived proteolytic metabolites p-cresol sulfate, p-cresol glucuronide, and phenylacetylglutamine according to enterotypes. Differences between enterotypes were detected using the Mann-Whitney *U* test. *p < 0.05, **p < 0.01, ***p < 0.001, ****p < 0.0001

Although slower intestinal transit would theoretically allow for more energy extraction, the stool energy density was, opposite of what would be expected, positively associated with intestinal transit time (Spearman rho = 0.23, *P* = 0.027; Supplementary Figure S2). Additionally, stratification of the individuals based on the abundance of the three established enterotype signature taxa [10] (Fig. 1b and Supplementary Figure S3) revealed significantly lower stool energy density in individuals with the *Bacteroides* (B-type) enterotype compared to individuals with the *Ruminococcaceae* (R type) enterotype and it also tended to be lower than in the P-type individuals (*P* = 0.06) (Fig. 1c). Bacterial genera correlating with the three signature taxa are provided in Additional file 2, and correlations between bacterial genera and stool energy density and transit time, respectively, are provided in Additional file 3. In agreement with the positive association between stool energy density and intestinal transit time, the B-type individuals had significantly shorter transit times compared to the R-type individuals, whereas the P-type individuals were in between (Fig. 1d). The differences in stool energy density among enterotypes were not explained by differences in habitual dietary patterns (Supplementary Figure S4) or bacterial cell counts in stool samples (Supplementary Figure S5). Instead, differences in stool energy density related to different microbiome ecosystem structures, since the microbiome alpha-diversity as assessed by Shannon index and observed richness differed significantly among enterotypes, with B-type as the least diverse and R-type as the most (Fig. 1e, f). In addition, stool energy density was positively associated with both observed richness and Shannon Index (Spearman rho = 0.32, *P* = 0.0037 and Spearman rho = 0.24, *P* = 0.030, respectively; Supplementary Figure S6). When examining this association within each of the enterotypes, the positive association between stool energy density and richness and Shannon Index was only evident within the B-type (Spearman rho = 0.51, *P* = 0.0042 and Spearman rho = 0.49, *P* = 0.0058, respectively), whereas this association was absent in the P and R-types (Supplementary Figure S6). Notably, the B-type individuals had higher body weight relative to the R-type individuals (Fig. 1g). To evaluate potential functional differences between enterotypes, faecal SCFAs and urinary levels of proteolytic host-microbial co-metabolites were compared between enterotypes. Generally, faecal SCFAs were not significantly correlated with stool energy density (Fig. 2a), and faecal levels of acetate, propionate and butyrate did not differ between enterotypes (Fig. 2b). However, higher levels of branched SCFAs (isobutyrate, 2-methylbutyrate, and isovalerate) were observed in faeces of the R-type individuals compared to the B-type individuals (Fig. 2c), whereas higher levels of valerate and caproate were observed in the P-type individuals compared to the B-type (Fig. 2d). Furthermore, very consistently, higher levels of microbial-derived proteolytic metabolites (p-cresol glucuronide, p-cresol sulfate, and phenylacetylglutamine) were observed in the urine of the R-type individuals compared with the B and P type individuals (Fig. 2e), suggesting differences in colonic proteolysis between enterotypes. Since assignment of enterotypes is non-trivial [12], we finally evaluated the robustness of the observed differences between enterotypes by excluding individuals (*n* = 28) for whom the assignment to an enterotype was ambiguous (i.e., positioned in the central part of the PCoA plot or in between more densely populated regions) (Fig. 1b and Supplementary Figure S7a). When analysing the subset of subjects, the observed differences between enterotypes remained robust, and statistical significances and effect sizes even increased despite the lower sample size (Supplementary Figure S7b–f).

## Discussion

Here, we show that gut microbiome structures depicted by enterotypes may differ in energy-extraction capacity, which could have implications for human energy balance and possibly explain weight gain variation [28]. Intriguingly, such hypothesis was tentatively supported by the finding that B-type individuals had a higher body weight compared to the R-type individuals (Fig. 1g). However, these body weight differences can not directly be linked to the stool energy density differences presented here, because the possible relations are complex and operate at a very different timescale. Nevertheless, the lower stool energy density and higher body weight of the B-type could suggest a more efficient energy extraction compared with the R- and P-types. The lower stool energy density of the B-type is consistent with a previous study showing that the B-type has higher metabolic capacity for both saccharolytic and proteolytic metabolism compared with the other enterotypes [14]. Indeed, previous studies have suggested that B-type individuals are less likely to lose body weight on fibre/wholegrain-rich diets relative to the P-type [29, 30]. The B-type has repeatedly been associated with a Western lifestyle low in microbiota-accessible carbohydrates, while the P-type has been associated with a fibre diet rich in MACs [31]. However, we did not observe any differences in habitual diet between the enterotypes, which may suggest that enterotypes are established earlier in life as previously suggested [32]. Instead, we found higher alpha-diversity and higher levels of microbiota-derived proteolytic metabolites in faeces and urine among the R-type individuals compared to the B and P type, suggesting a more complex microbial ecosystem with increased colonic proteolysis in the R-type individuals. This could possibly be explained by the longer transit time in the R-type individuals, since we and others have previously shown that a long intestinal transit time is associated with both alpha-diversity and increased proteolytic fermentation [16, 17], whereas a short transit time is associated with increased saccharolysis [33]. Here, we did also observe negative correlations between transit time and faecal acetate/propionate/butyrate, however no differences were found in these metabolites among enterotypes. While the P and B-type repeatedly have been identified due to the bi-modal distribution of *Prevotella* [12], our study suggests that the less clear separation between R and B types may be driven by a gradient in transit time (Fig. 1b, d). Here, we also extend previous findings by showing that transit time is positively associated with stool energy density. Taken together, this suggests that a long intestinal transit time does not necessarily lead to more complete dietary substrate-depletion, but is accompanied by a shift in microbial fermentation from saccharolytic to proteolytic metabolism, which negatively affects the gut microbiota’s energy extraction leading to less complete dietary substrate-depletion as reflected by the higher stool energy density. This questions the general notion that carbohydrates are depleted in individuals with a long transit time. While the general notion is mostly based on depletion of readily accessible carbohydrate resources [34], we hypothesise that more complex carbohydrates derived from food may not effectively be degraded into readily fermentable simple carbohydrates by the R-type ecosystem, whereby that ecosystem switches to proteolytic energy generation making carbohydrate depletion incomplete. Our findings are in apparent agreement with a previous study that demonstrated that slowing transit time in human volunteers reduced the saccharolytic activity of the faecal donor material, as reflected by significantly decreased short-chain fatty acid production and dietary fibre degradation upon in vitro fermentation [35].

**Fig. 3:**
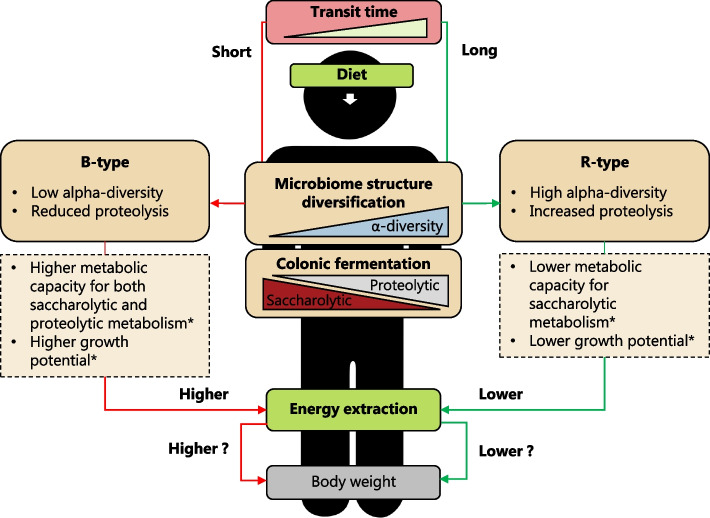
Proposed human gut microbiota-dependent energy extraction. We propose that intestinal transit time diversifies the gut microbiome community structures into preferred community structures captured by enterotypes (B-type, *Bacteroides*; R-type, *Ruminococcaceae*; and P-type, *Prevotella*) with the B-type and R-type being most distinct in terms of transit time and alpha-diversity. The B and R-type enterotypes differ in alpha-diversity and colonic fermentation, which in essence is the trade-off between saccharolytic and proteolytic metabolism, which may affect the enterotypes’ overall efficiency to extract energy from food. This could potentially translate into different body weight. The stars (*) refer to findings from a previously published paper showing that enterotypes differ in metabolic capacity and growth potential [14]

Although the present observational study was not designed to reveal causal relationships, we suggest that the intestinal transit time diversifies the gut microbiome community structures and thereby influence the overall efficiency of the gut microbiome to extract energy from food (Fig. 3). This raises important questions about the ecological foundations underlying these relationships. Previous functional analysis of the enterotypes, led to the proposition that the B-type reflects a *r*-selection enriched ecosystem (fast growth and squandering substrate utilisation) compared to the more *K*-selected and functionally redundant R-type (slow growth and high substrate-energy efficiency) [14, 36], which agrees with the higher estimated average microbial growth rate of the B-type compared to the R-type [14]. However, higher relative abundance of *K*-strategists in the R-type ecosystem would predict that it would display higher energy extraction capacity compared to the B-type, which is in contrast to what we observe in the energy density of the stool samples. This apparent contradiction requires further deciphering of driving forces that shape the gut microbial ecosystem. The present study has several limitations. First of all, it was not designed to investigate links between microbiome compositions and stool energy density. Furthermore, due to the inherit limitations of dietary assessment tools, potentially confounding factors such as energy intake could not be corrected for. Therefore, future studies should test the proposed hypothesis in well-controlled settings controlling for total calories intake and excretion. This would allow the assessment of stool energy excretion and not only stool energy density. Furthermore, apart from the bacterial counts in faeces, it was not possible in the present study to investigate the composition of the remaining solid residual faeces. Finally, with the limited sample size, we were not able to adjust for potential confounders such as gender.

## Conclusions

In conclusion, our study demonstrates that stool energy density, as a proxy of gut microbial energy harvest, is associated with intestinal transit time and microbiome community structures. The study offers some of the first evidence to suggest that differences in human gut microbial community structures as reflected by enterotypes affect the gut microbiota’s ability to extract energy from food. While the causalities remain to be established, the observations are intriguing and could be pivotal for predicting personalised dietary responses in body weight management.

## Supplementary Information

**Additional file 1.**

**Additional file 2.**

**Additional file 3.**

**Additional file 4: Supplementary Figure S1.** Flow chart of study participants and available data. Baseline measurements from participants of two human intervention studies that were carried out at the same site were included in the present study. In total, 85 adults were included where measurements on transit time, gut microbiome, urine metabolites, and habitual diet were available. Stool aliquots were analysed for stool energy density (n=80), stool bacterial counts (n=83), and faecal short-chain fatty acids (SCFA, n=83). Stool energy density outliers (n=3) were identified using the ROUT method with a maximum desired false discovery rate set to 1%. **Supplementary Figure S2.** Association between intestinal transit time and stool energy density. Spearman Rank correlations between intestinal transit time and stool energy density. The lines show linear regressions with 95% confidence bands of the best-fit line indicated with the grey shading between the dotted lines. **Supplementary Figure S3.** Stratification according to microbial enterotypes. (a) Relative abundances of the three characteristic bacterial taxa contributing to each of the three enterotypes. The box plots show the lower and upper quartiles and the median with the whiskers indicating the minimum and maximum abundances. Red: *Bacteroides* (B-type), n=35; yellow: *Prevotella* (P-type), n=16; green: *Ruminococcaceae* (R-type), n=34. (b) Principal coordinate analysis plot using Bray-Curtis distance of bacterial relative abundance on the genus level as distance metric. Symbols are samples, with shape / colour indicating assigned enterotype (red circles: B-type; yellow diamonds: P-type; green squares: R-type). All genera with an average relative abundance of 0.1% or more (21 in total) were plotted as supplementary variables (back arrows). OTUs that could not be assigned up to the genus level were assigned to a dummy “genus” labelled with the most specific classification available for that OTUs. The first letter of each arrow indicated this level (g: genus, f: family, o: order). **Supplementary Figure S4.** Enterotypes did not differ in habitual diet. (a) Principal component analysis on dietary data (g/day/kJ) with particiants (n=85) colored according to enterotype in the scores plot. (b) Furthermore, no differences were found between enterotypes in dietary pattern when comparing intake of macronutrients and different food groups and components. Differences between enterotypes were assessed using the Mann-Whitney U test. **Supplementary Figure S5.** No differences among enterotypes in stool bacterial cell counts. Subjects stratified into enterotypes did not differ in bacterial cell counts in stool samples (n=83). Differences between enterotypes were assessed using the Mann-Whitney U test. ns, not significant. **Supplementary Figure S6.** Associations between stool energy density and microbiome alpha-diversity. Spearman Rank correlations between stool energy density and microbiome alpha diversity as assessed by (a) observed richness and (b) Shannon Index, respectively. Individuals are coloured according to their microbial enterotype designation and the enterotypes-specific Spearman rank correlations are shown next to the graphs. The lines show linear regressions with 95% confidence bands of the best-fit regression line. **Supplementary Figure S7.** Re-analyses of enterotype differences in subset of subjects. (a) To evaluate the robustness of the observed differences between enterotypes, individuals for whom the assignment to an enterotype was ambiguous (i.e., positioned in the central part of the principal coordinate analysis (PCoA) plot or in between more densely populated regions) were excluded (n=28). The PCoA plot was generated using Bray-Curtis distance of bacterial relative abundance on the genus level as distance metric. Symbols are samples, with shape / colour indicating assigned enterotype (red circles: *Bacteroides* (B-type), n=21; yellow diamonds: *Prevotella* (P-type), n=15; green squares: *Ruminococcaceae* (R-type), n=21; grey dots: unclassified, n=28). Relative abundances of the taxa used for enterotype assignment (black arrows) were plotted supplementary (i.e., projected after ordination). Horizontal and vertical axis explain 20% and 12% of variation, respectively. The subset of subjects stratified into three enterotypes differed in (b) stool energy density (n=51), (c) intestinal transit time (n=57), microbiome alpha-diversity as reflected by (d) Shannon Index and (e) observed richness (n=57), and (f) body weight (n=57). Differences between enterotypes were detected using the Mann-Whitney U test. * p < 0.05, ** p < 0.01, *** p<0.001, **** < 0.0001.

## Data Availability

Sequence analysis data were deposited in the NCBI SRA database under accession no. SRP070699. The remaining data that were included, generated and analysed during the current study are included with this article as a supplementary data file (Additional file 1).

## Abbreviations

B-type: *Bacteroides* enterotype
P-type: *Prevotella* enterotypes
R-type: *Ruminococcaceae* enterotype
PCoA: Principal coordinates analysis
