# Supplementary material for: Stool energy density is positively correlated to intestinal transit time and related to microbial enterotypes
Source: Microbiome. 2022 Dec 12;10:223. doi: 10.1186/s40168-022-01418-5 (PMC9743556; doi:10.1186/s40168-022-01418-5)
Supplement: Supplementary file 5 — Additional file 4: Supplementary Figure S1. Flow chart of study participants and available data. Baseline measurements from participants of two human intervention studies that were carried out at the same site were included in the present study. In total, 85 adults were included where measurements on transit time, gut microbiome, urine metabolites, and habitual diet were available. Stool aliquots were analysed for stool energy density (n=80), stool bacterial counts (n=83), and faecal short-chain fatty acids (SCFA, n=83). Stool energy density outliers (n=3) were identified using the ROUT method with a maximum desired false discovery rate set to 1%. Supplementary Figure S2. Association between intestinal transit time and stool energy density. Spearman Rank correlations between intestinal transit time and stool energy density. The lines show linear regressions with 95% confidence bands of the best-fit line indicated with the grey shading between the dotted lines. Supplementary Figure S3. Stratification according to microbial enterotypes. (a) Relative abundances of the three characteristic bacterial taxa contributing to each of the three enterotypes. The box plots show the lower and upper quartiles and the median with the whiskers indicating the minimum and maximum abundances. Red: Bacteroides (B-type), n=35; yellow: Prevotella (P-type), n=16; green: Ruminococcaceae (R-type), n=34. (b) Principal coordinate analysis plot using Bray-Curtis distance of bacterial relative abundance on the genus level as distance metric. Symbols are samples, with shape / colour indicating assigned enterotype (red circles: B-type; yellow diamonds: P-type; green squares: R-type). All genera with an average relative abundance of 0.1% or more (21 in total) were plotted as supplementary variables (back arrows). OTUs that could not be assigned up to the genus level were assigned to a dummy “genus” labelled with the most specific classification available for that OTUs. The first letter of each arro [file 40168_2022_1418_MOESM4_ESM.docx]

**SUPPLEMENTARY INFORMATION**

**Supplementary Figures**

**
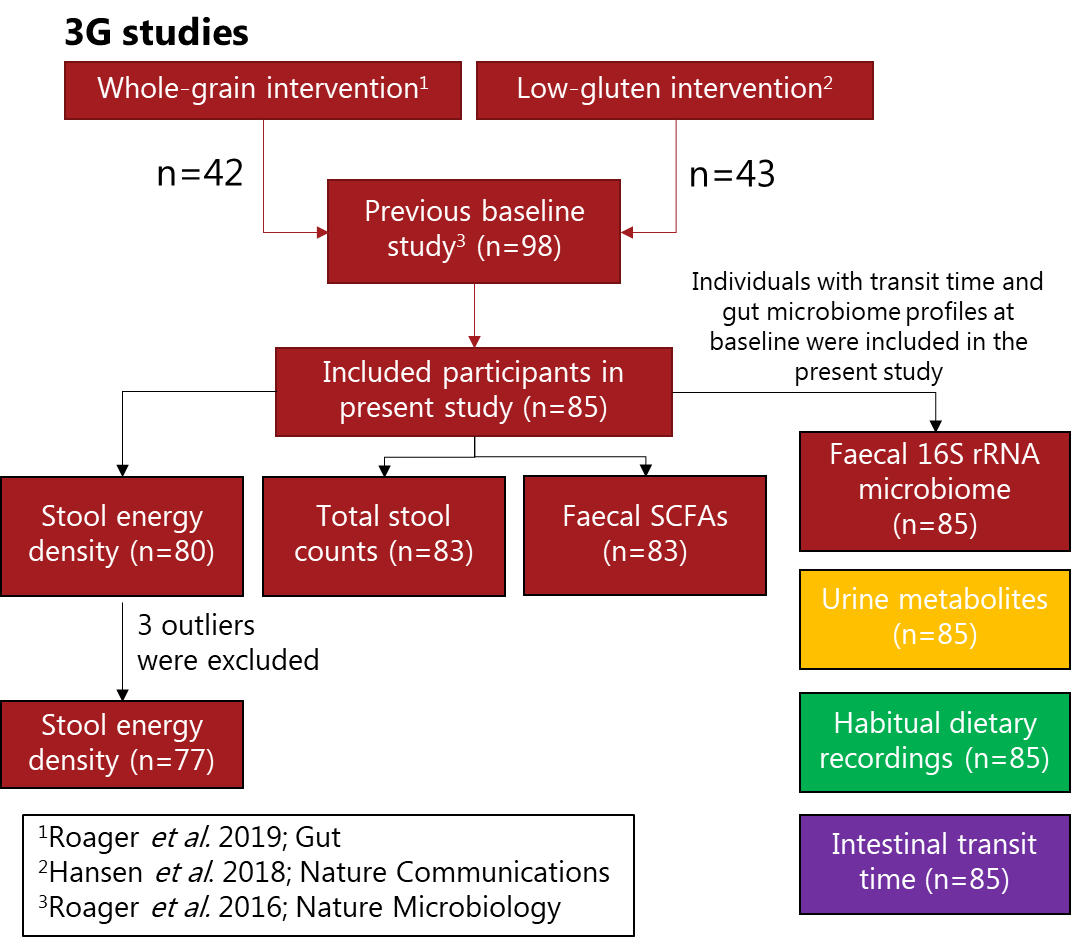
**

**Supplementary Fig 1. Flow chart of study participants and available data**Baseline measurements from participants of two human intervention studies that were carried out at the same site were included in the present study. In total, 85 adults were included where measurements on transit time, gut microbiome, urine metabolites, and habitual diet were available. Stool aliquots were analysed for stool energy density (n=80), stool bacterial counts (n=83), and faecal short-chain fatty acids (SCFA, n=83). Stool energy density outliers (n=3) were identified using the ROUT method with a maximum desired false discovery rate set to 1%.

**
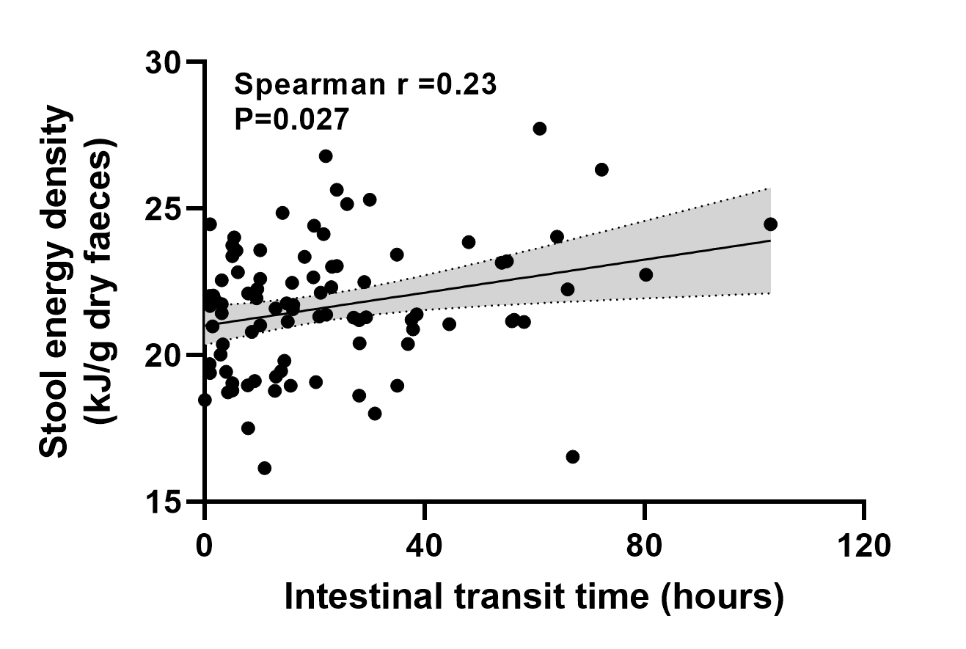
**

**Supplementary Fig 2. Association between intestinal transit time and stool energy density**. Spearman Rank correlations between intestinal transit time and stool energy density. The lines show linear regressions with 95% confidence bands of the best-fit line indicated with the grey shading between the dotted lines.

**Supplementary Fig 3. Stratification according to microbial enterotypes. (a)** Relative abundances of the three characteristic bacterial taxa contributing to each of the three enterotypes. The box plots show the lower and upper quartiles and the median with the whiskers indicating the minimum and maximum abundances. Red: *Bacteroides* (B-type), n=35; yellow: *Prevotella* (P-type), n=16; green: *Ruminococcaceae* (R-type), n=34. (**b**) Principal coordinate analysis plot using Bray-Curtis distance of bacterial relative abundance on the genus level as distance metric. Symbols are samples, with shape / colour indicating assigned enterotype (red circles: B-type; yellow diamonds: P-type; green squares: R-type). All genera with an average relative abundance of 0.1% or more (21 in total) were plotted as supplementary variables (back arrows). OTUs that could not be assigned up to the genus level were assigned to a dummy “genus” labelled with the most specific classification available for that OTUs. The first letter of each arrow indicated this level (g: genus, f: family, o: order).

**Supplementary Fig 4. Enterotypes did not differ in habitual diet**. (**a**) Principal component analysis on dietary data (g/day/kJ) with particiants (n=85) colored according to enterotype in the scores plot. (**b**) Furthermore, no differences were found between enterotypes in dietary pattern when comparing intake of macronutrients and different food groups and components. Differences between enterotypes were assessed using the Mann-Whitney U test.

**Supplementary Fig 5. No differences among enterotypes in stool bacterial cell counts**Subjects stratified into enterotypes did not differ in bacterial cell counts in stool samples (n=83). Differences between enterotypes were assessed using the Mann-Whitney U test. ns, not significant.

**
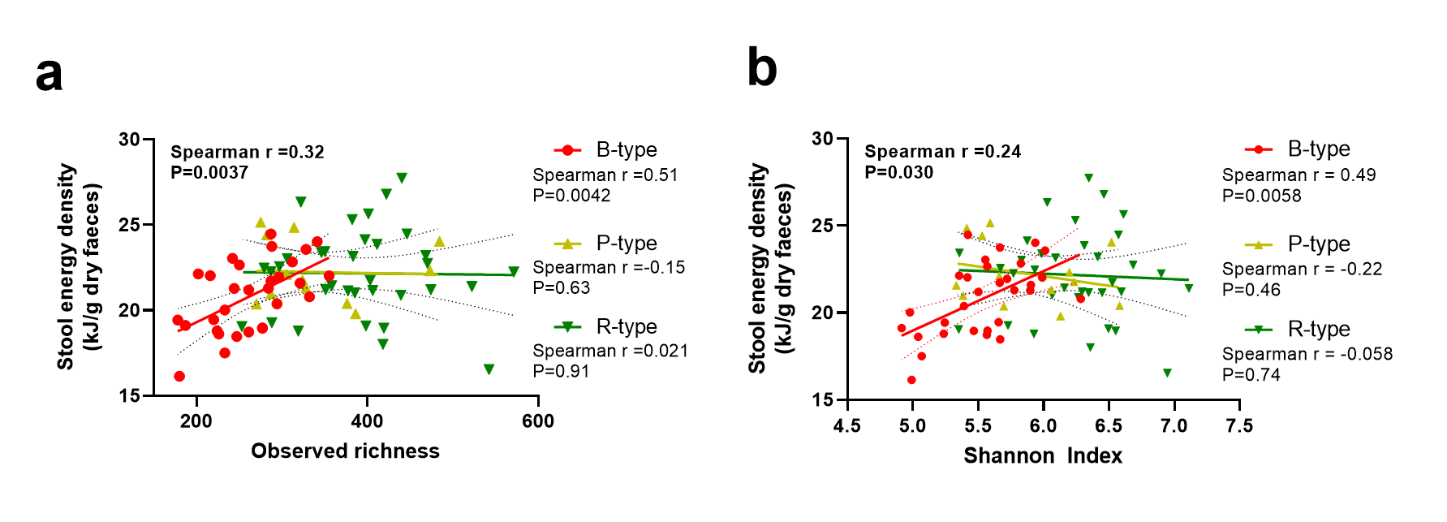
**

**Supplementary Fig 6. Associations between stool energy density and microbiome alpha-diversity**. Spearman Rank correlations between stool energy density and microbiome alpha diversity as assessed by (**a**) observed richness and (**b**) Shannon Index, respectively. Individuals are coloured according to their microbial enterotype designation and the enterotypes-specific Spearman rank correlations are shown next to the graphs. The lines show linear regressions with 95% confidence bands of the best-fit regression line.


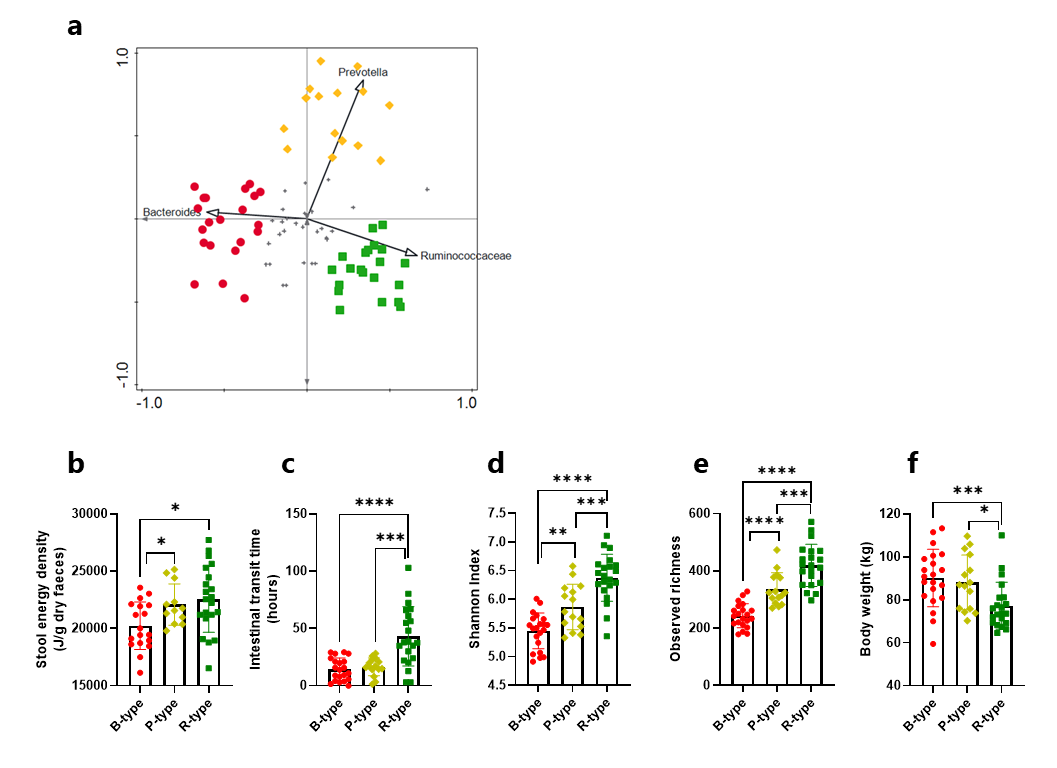


**Supplementary Fig 7. Re-analyses of enterotype differences in subset of subjects.** (**a**) To evaluate the robustness of the observed differences between enterotypes, individuals for whom the assignment to an enterotype was ambiguous (i.e., positioned in the central part of the principal coordinate analysis (PCoA) plot or in between more densely populated regions) were excluded (n=28). The PCoA plot was generated using Bray-Curtis distance of bacterial relative abundance on the genus level as distance metric. Symbols are samples, with shape / colour indicating assigned enterotype (red circles: *Bacteroides* (B-type), n=21; yellow diamonds: *Prevotella* (P-type), n=15; green squares: *Ruminococcaceae* (R-type), n=21; grey dots: unclassified, n=28). Relative abundances of the taxa used for enterotype assignment (black arrows) were plotted supplementary (i.e., projected after ordination). Horizontal and vertical axis explain 20% and 12% of variation, respectively. The subset of subjects stratified into three enterotypes differed in (**b**) stool energy density (n=51), (**c**) intestinal transit time (n=57), microbiome alpha-diversity as reflected by (**d**) Shannon Index and (**e**) observed richness (n=57), and (**f**) body weight (n=57). Differences between enterotypes were detected using the Mann-Whitney U test. * p < 0.05, ** p < 0.01, *** p<0.001, **** < 0.0001.
